# Establishment of a clinical cancer genetics program for breast cancer in a resource-limited country; challenges and opportunities

**DOI:** 10.3389/fonc.2024.1431985

**Published:** 2024-10-23

**Authors:** Hikmat Abdel-Razeq, Baha Sharaf, Faris Tamimi, Hira Bani Hani, Osama Alsmadi, Hanan Khalil, Mahmoud Abunasser, Sarah Edaily, Asem Mansour

**Affiliations:** ^1^ Department of Internal Medicine, King Hussein Cancer Center, Amman, Jordan; ^2^ School of Medicine, The University of Jordan, Amman, Jordan; ^3^ Department of Cell Therapy and Applied Genomics, King Hussein Cancer Center, Amman, Jordan; ^4^ Department of Radiology, King Hussein Cancer Center, Amman, Jordan

**Keywords:** clinical cancer genetics program, BRCA, inherited breast cancer, low-income countries, germline mutation

## Abstract

Breast cancer is the most common cancer among women worldwide, and its incidence rate is still increasing, especially among younger women. Nationally, it constitutes one-fifth of all cancer cases and almost 40% of all female cancers. With a median age of 51 years, breast cancer is diagnosed at least a decade earlier, and at more advanced stages compared to Western societies. Hereditary cancers account for 10% or more of all cancer burden worldwide. With expanded indications, increased number of genes tested, and significant decline in cost of testing, such proportion will probably increase. Individuals with pathogenic variants of *BRCA1* and *BRCA2* are at higher risk of breast, ovarian, pancreatic and many other cancers. Over the past two decades, several highly penetrant cancer-susceptibility genes were identified across almost all tumor sites, thus increasing the need for comprehensive cancer genetic programs that address the testing process, counselling patients and at-risk family members, and then deal with all testing results and its consequences. In addition to its important role in preventing more cancers in index patients themselves and among their close relatives, identification of pathogenic or likely pathogenic variants, mostly in *BRCA1* or *BRCA2*, may inform therapeutic decisions in common cancers including breast, ovarian, prostate and pancreatic cancers. In this manuscript, we describe the experience of a comprehensive cancer center, in a resource-limited country in establishing a comprehensive clinical cancer genetics program that can serve as an example for others who share similar demographic and financial restrains.

## 1 Introduction

Jordan is a small country (89,000 square km) located in the west part of the Middle East. The population has grown recently to over 11 million, affected mostly by the influx of refugees from neighboring countries (1). Much of the population is heavily concentrated around Amman, the capital, and in the northwest cities. Population of Jordan is relatively young, with a median age of 22.5 years and only 3.5% are 65 years and older (2). Jordan’s economy is among the smallest in the region with insufficient supplies of water and limited natural resources. High rate of unemployment, especially among younger individuals, adds to the economic challenges (3). The country is classified by the World Bank as a “lower middle-income country (LMIC)” with a Gross Domestic Product (GDP) of $48.65 billion and $4311.0 per capita (4).

King Hussein Cancer Center (KHCC) is a 352-bed stand-alone tertiary cancer center established in 2001 to serve Jordanians and patients from the region. The center covers cancer care dimensions across the continuum including screening and early detection, cancer prevention, active treatment, palliative/hospice care and survivorship. Additionally, the center is actively engaged in clinical research and serves as the regional hub for cancer-related medical training and education (5, 6).

Breast cancer is the most common cancer among women worldwide (7, 8). Recent data have suggested that the incidence rate of breast cancer increased during 2015-2019 by almost 1% annually (6, 7). Nationally, over 1750 new breast cancer cases were diagnosed and reported by the Jordan Cancer Registry in 2022. As such, it constitutes one-fifth of all cancer cases and almost 40% of all female cancers. Additionally, one fourth of all cancer-related mortality in women are due to breast cancer (9). With a median age of 50-52 years, breast cancer in Jordan, and most of the Arab world, is diagnosed at least a decade earlier compared to the West. To complicate the issue further, more than a third of patients present late with metastatic or locally advanced disease (10, 11).

## 2 Hereditary cancers

Hereditary cancers account for 10% or more of cancer burden worldwide (12). With our improving understanding of molecular biology, wider access to diagnostic technology and expanded indications for testing, such proportion will significantly increase. Over the past two decades, several highly penetrant cancer-susceptibility genes were identified, thus establishing the nidus for a new dimension in counselling and cancer prevention (13, 14). Additionally, identification of pathogenic or likely pathogenic (P/LP) variants may inform therapeutic decisions in common cancers including breast, ovarian, prostate and pancreatic cancers (15, 16).

Individuals with pathogenic variants of *BRCA1* and *BRCA2* are at higher risk of breast and ovarian cancers (17, 18). In one study that prospectively included a cohort of 978 *BRCA1* and 909 *BRCA2* pathogenic carriers from the United Kingdom, the average cumulative risks by age 70 years for *BRCA1* carriers were estimated to be 60% (95% confidence interval [CI], 44%-75%) for breast cancer and 59% (95% CI, 43%-76%) for ovarian cancer. Women with *BRCA2* pathogenic variants had a corresponding risk of 55% (95% CI, 41%-70%) and 16.5% (95% CI, 7.5%-34%) for breast and ovarian cancers, respectively (19). The estimated risk for contralateral breast cancer is 83% (95% CI, 69%-94%) for *BRCA1* carrier and 62% (95% CI, 44%-79.5%) for *BRCA2.* A meta-analysis of ten eligible studies that looked at the penetrance rates of *BRCA1* and *BRCA2* reached similar conclusions (20).

Despite the undebatable importance of germline genetic testing, access and uptake of such services is limited and mostly dependent on financial and psychosocial structure of particular society or country.

## 3 Genetic testing

### 3.1 The start: from research to clinical practice

The process of genetic counselling and testing at KHCC started as a research project in 2016. Through a competitive grant from KHCC and MD Anderson Cancer Center Sister Institution Network Fund (SINF), we enrolled 100 young Jordanian breast cancer women (median age, 40 years) who were considered at higher risk of harboring a germline variant and testing was carried at a commercial lab (Myriad Genetics, Salt Lake City, Utah). In total, 27 (27.0%) patients had pathogenic or likely pathogenic *BRCA1* or *BRCA2* variants; the only two variants were tested at that time. Higher mutation rates were observed among patients with triple negative disease and those with positive family history of breast or ovarian cancers (21). Giving the research setup under which the genetic testing was performed, the process was highly regulated and monitored by our Institutional Review Board (IRB) which enhanced our capabilities to convert the testing process to a clinical service. Several issues including patients’ consenting, pre- and post-genetic testing counselling, communication with close relatives and cascade testing were all improved.

**Figure 1:**
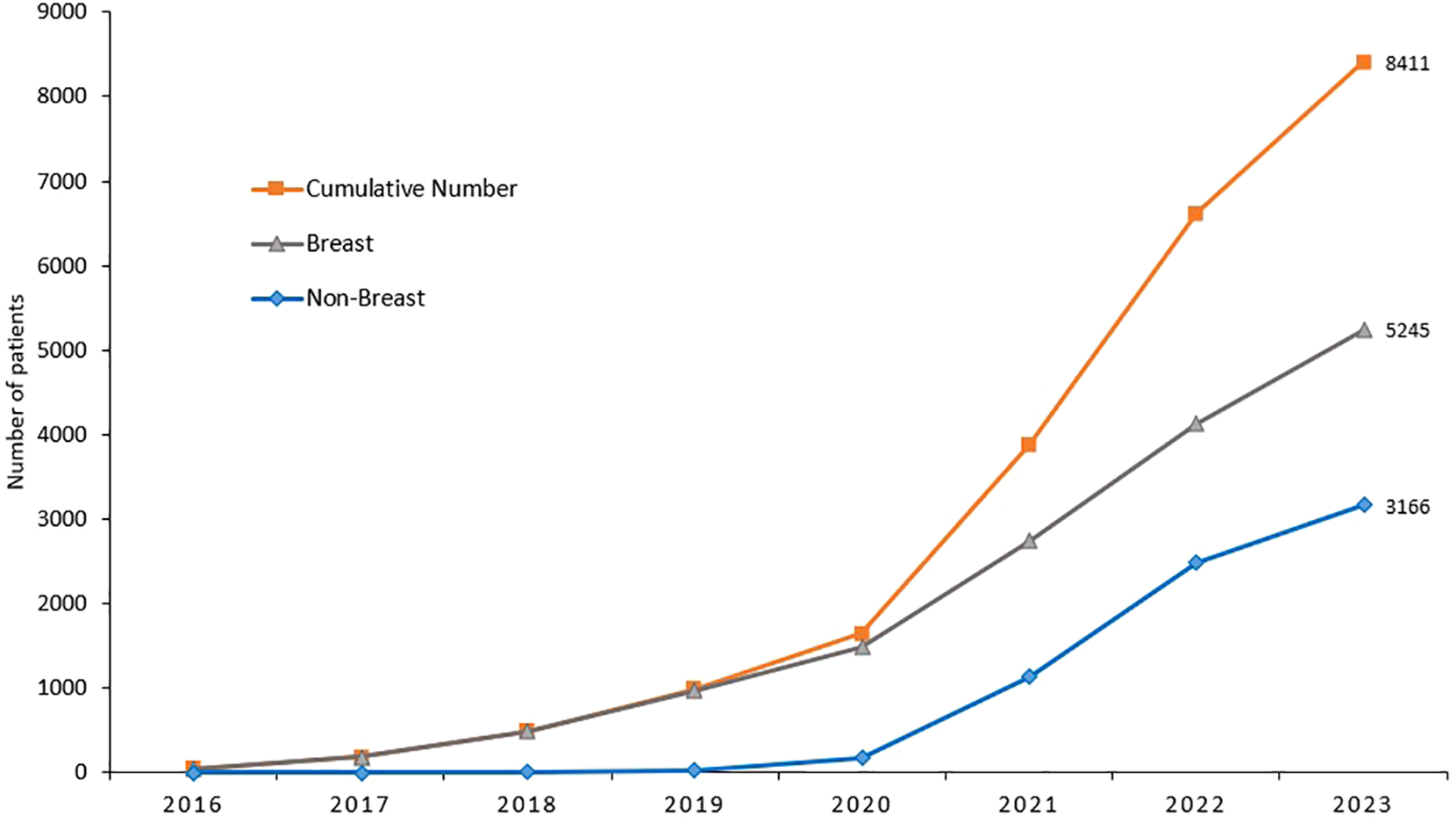
Germline genetic testing among patients with all primary sites and breast cancer.

**Figure 2:**
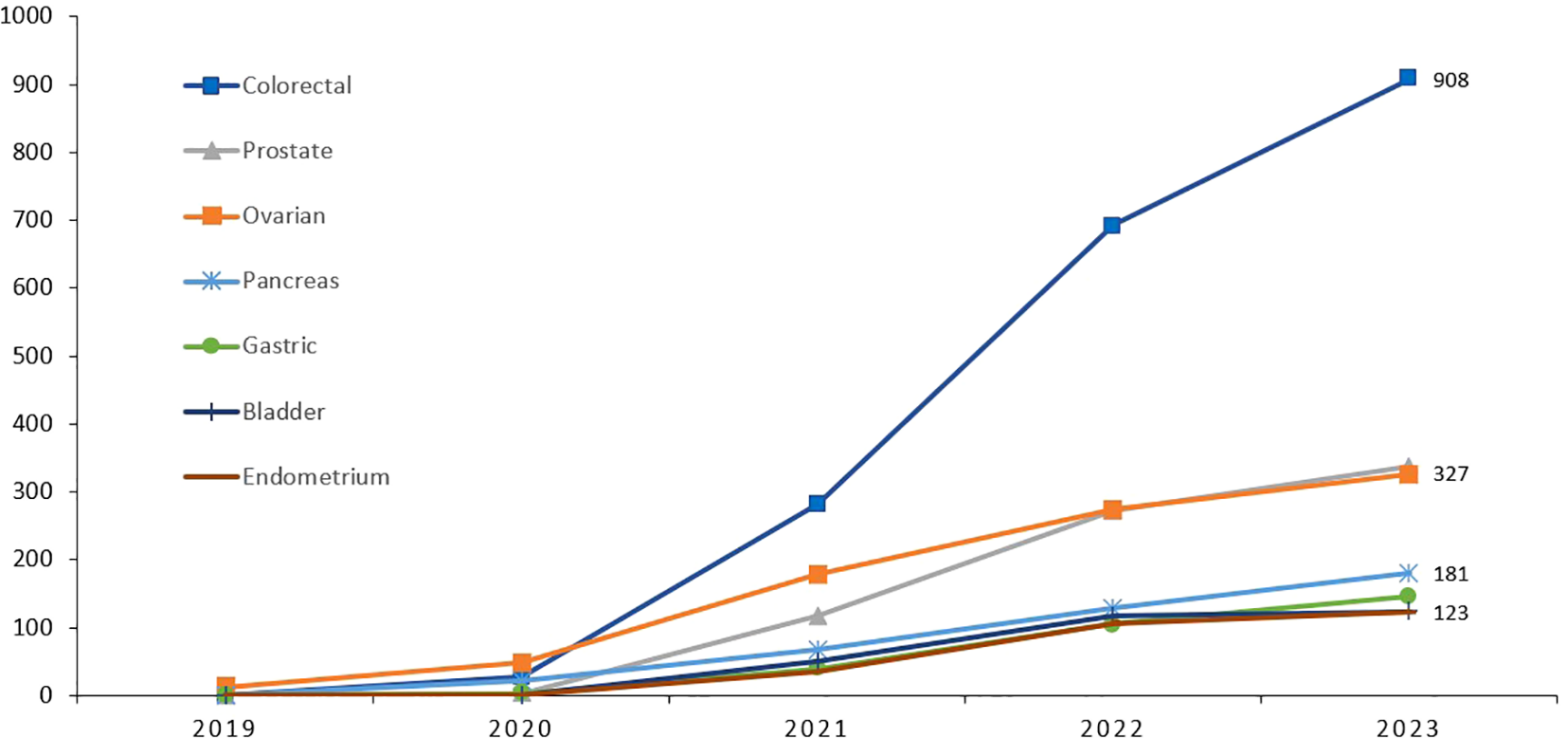
Cumulative number of patients with cancers other than breast, who had germline genetic testing illustrating the significant increase in uptake.

Though the cost of genetic testing has declined significantly over the past few years, access can still be a financial burden. Recent advances in molecular diagnostics and its clinical implications on cancer management have put lots of pressure on resource-limited countries. Soon after the completion of our study, genetic testing and counselling are routinely practiced in our center across all primary tumor sites, not only breast cancer, and according to international guidelines. Cost of germline genetic testing is mostly covered by cancer-specific insurance. Since we started, over 9,000 cancer patients were tested; over half of them are breast cancers, **Figures 1**, **2**. It’s important to highlight here that very few patients refuse germline genetic testing, both for research purposes or when clinically indicated. However, the uptake of cascade testing for at-risk “healthy” relative is lower than anticipated. Cost of testing and potential risk-reducing interventions continue to be important barriers.

Such “comprehensive” service is not readily available elsewhere in the country for almost half of cancer patients treated outside our institution and when performed, it’s only the testing part and mostly without proper pre- or post-testing counselling and mostly without cascade testing for at-risk family members. Patients’ referral to centers where such service is provided is not easy; logistics related to insurance coverage and communication with patients themselves and close relatives are among the main issues encountered.

### 3.2 Eligible patients

We follow the National Comprehensive Cancer Network (NCCN) and other international guidelines, including the American Society of Clinical Oncology (ASCO), for germline genetic testing (22–24). These guidelines are frequently updated and not all physicians, including medical and surgical oncologists, are familiar with such very frequent updates; a factor that may contribute to the lower referral of eligible patients for testing and counselling. To enhance referral rate, germline genetic testing was included as a KPI (Key Performance Indicator) and was added to the discussion points during our weekly multidisciplinary team meetings of each new breast cancer patient. Over the past few years, the age at which the NCCN recommends commence testing, regardless of personal or family history of cancer, was raised from 40 years, to 45, then 50, and more recently was raised to 65 by the ASCO and the Society of Surgical Oncology (ASO). Current indications for genetic testing are summarized in **Table 1**.

**Table 1:** Indications for germline genetic testing*.

Age
▪ All patients aged ≤65 years regardless of personal or family history or tumor characteristics
Gender
▪ All male patients with breast cancer
Ethnicity:
▪ All Patients with Ashkenazi Jewish ancestry
Pathology
▪ All patients with triple-negative breast cancer
▪ All patients with multiple primary breast cancers (synchronous or metachronous)
▪ All patients with Invasive lobular carcinoma (ILC) with personal or family history of diffuse gastric cancer
Family history
▪ All patients with close blood relative with:
▪ Breast cancer at age ≤50 years
▪ Male breast cancer
▪ Ovarian cancer
▪ Pancreatic cancer
▪ Prostate cancer (metastatic or high-risk )
Patients > 65 years, may be tested if:
▪ Patients with early-, or advanced-stage disease who are candidates for poly (ADP–ribose) polymerase (PARP) inhibitors.
▪ Patients who develop a second primary cancer in the ipsilateral or contralateral breast
▪ Positive family history
▪ Patients with personal or family history that suggest the possibility of a pathogenic variant

*Adopted from the National Comprehensive Cancer Network (NCCN) (22) and the American Society of Clinical Oncology (ASCO) (23, 24).

Though we advocate using guidelines-based testing, some clinicians and researchers advocate universal testing of all women with breast cancer. Such a new direction is supported by several recent publications that showed higher rates of missed opportunities, should we restrict testing to those suggested by the guidelines (25–27). The American Society of Breast Surgeons endorsed this universal testing approach (28).

The new expanded testing guidelines will probably include over 80% of patients with breast cancer. Given the younger age at breast cancer diagnosis, and the very low percentage of Jordanians above the age of 65 years (less than 5%), we expect that current guidelines would cover over 90 or 95% of all newly diagnosed breast cancer patients.

### 3.3 Variants of uncertain significance

At the initial phases of setting up our program, genetic testing was restricted to *BRCA1* and *BRCA2* which was expanded initially to include *PALB2* and *CHEK2*. However, currently we test for 19 genes (*ATM, BARD1, BRCA1, BRCA2, BRIP1, CDH1, CHEK2, EPCAM, MLH1, MSH2, MSH6, NF1, PALB2, PMS2, PTEN, RAD51C, RAD51D, STK11, TP53*). With expanded gene testing, we faced the problem with high percentage of Variants of Uncertain Significance (VUS) which reached rates beyond 50%. Such high rates can be explained by the under representation of “Arabs” in international genetic variants libraries (29). As more information becomes available, some of the VUS can be modified and upgraded to “pathogenic”. Though few, so far, such changes put lots of pressure on health care systems to be able to communicate such upgrades with patients tested months or even years later. To help avoid any mishaps in communicating these results, and its associated clinical decisions, we involve the patients themselves and recommend they also check with their health care providers with subsequent follow up visits; a statement that is included in the consent form, too.

### 3.4 Genetic counselling

There isn’t any formal educational or training programs in the country that graduate genetic counselors. As such, medical and surgical oncologists were the ones who started the process of pre- and post-testing counselling. However, as we can imagine, time needed for this process is difficult to allocate in a busy schedule of a surgeon or a medical oncologist. To help solve this problem, we managed to train health care workers with basic education in molecular biology, pharmacy or nursing, to become “clinical counsellors”. Currently, 3 genetic counselors are running daily “genetic counseling clinics” independently. However, and given the increasing number of patients tested, we also established cancer genetics clinics that are run daily by medical oncologists; some were trained at one of our international collaborative institutions. Our hybrid approach (counselors and medical oncologists) is unique and can serve as an example of a multidisciplinary approach, especially in resource-limited countries.

## 4 Local data

In one of our studies, a total of 1310 patients with breast cancer were tested as per the NCCN guidelines. Age ≤ 45 years was the most common indication for testing (n= 800, 61.1%), while positive family history of breast, ovarian, pancreatic or prostate cancers, and triple-negative disease were among other common indications. Among the whole group, 184 (14.0%) patients had P/LP variants with only 90 (48.9%) were in *BRCA1* or *BRCA2*, while the others had pathogenic variants in other genes including *APC, TP53, CHEK2 and PALB2.* Variants of uncertain significance (VUS) were reported in 559 (42.7%) patients; majority (90.7%) were in genes other than *BRCA1* or *BRCA2* (30).

In another study that involved 616 younger patients diagnosed with breast cancer at age 40 or younger; 75 (12.2%) patients had P/LP variants; two of the *BRCA2* mutations were novel. In multivariate analysis, triple−negative disease (Odd Ratio [OR]: 5.37; 95% CI, 2.88–10.02, P< 0.0001), family history of breast cancer in ≥ 2 family members (OR: 4.44; 95% CI, 2.52–7.84, P< 0.0001), and a personal history ≥ 2 primary breast cancers (OR: 3.43; 95% CI, 1.62–7.24, P= 0.001) were associated with higher mutation rates (31).

**Figure 3:**
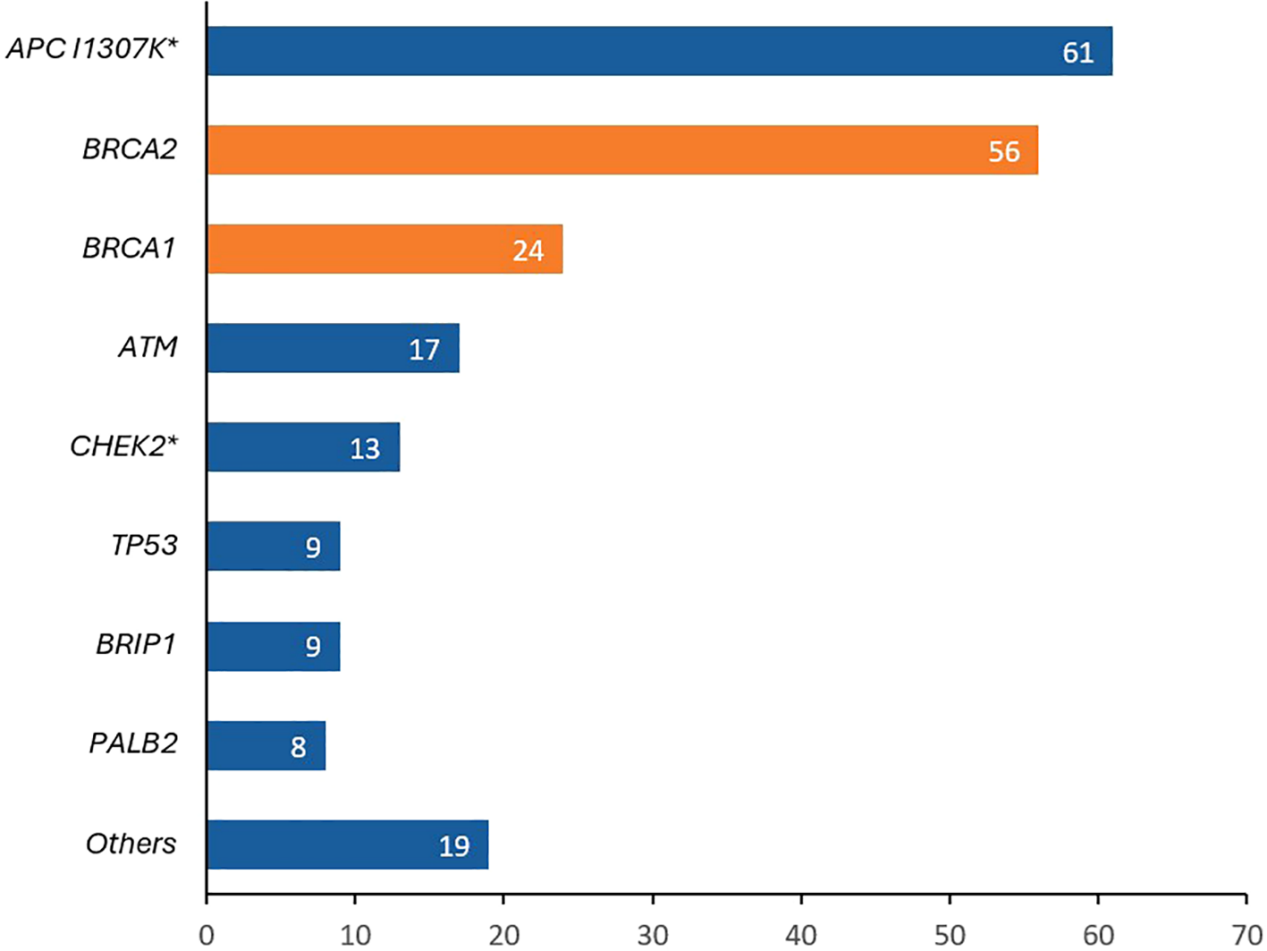
Pathogenic/likely pathogenic (P/LP) variants detected in patients with breast cancer patients tested as part of a universal germline genetic testing program. *APC I1307K* was the most identified variant (more common than *BRCA1* and *BRCA2*). *8 patients had biallelic APC I1307K, 1 patient had a biallelic CHEK2 and 1 patient had a biallelic PMS2.

More recently, our group enrolled a total of 3,319 unselected patients with solid tumors, regardless of their personal or family history of cancer on a universal cancer germline genetic testing study, 1672 (50.4%) had breast cancer. Patients were classified into two groups; those who met the NCCN 2020 testing criteria and those who did not. Pathogenic germline variants were detected in 14.5% of the 1125 breast cancer who met the testing criteria compared to 8.0% of 547 patients who did not. Common variants identified are summarized in **Figure 3** (32).

## 5 Cascade testing

Following the establishment of testing guidelines across all primary cancer sites, the identification of the index case is never an issue. Patients’ pre-testing assessment includes a three-generation family tree and at-risk family members are identified by the genetic counselor. Patients with positive germline mutation are asked to communicate such results with at-risk relatives. Appointments with the genetic counselling clinic, and occasionally with the primary oncologist, are arranged. Those who wish to be tested, after appropriate consenting, are offered the test almost at no-cost, if performed within 90 days of the index case. The uptake rate for cascade testing was low but improving.

Additionally, we face significant obstacles in dealing with positive pathogenic mutation among unaffected carriers, too. In one observational cross-sectional study performed by our group to assess level of communication between patients and their at-risk relatives, 169 breast cancer patients with positive breast cancer-susceptibility germline variants, were invited to participate. The cohort included 42 (24.9%) with P/LP *BRCA1* variants, 84 (49.7%) with *BRCA2* and 43 (25.4%) with non-BRCA variants. Results of P/LP variants were communicated with family members by majority (n= 160, 94.7%), including 642 first degree female relatives, however, 286 (44.5%) of them have taken no actions. Fear of positive test results, cost of testing, unwillingness to undergo preventive measures, and social stigma were cited as barriers to genetic testing in 54%, 50%, 34% and 15%, respectively (33).

Extensive work is needed to increase awareness of patients and relatives about the associated risk and high impact of risk-reducing interventions. However, such awareness campaigns won’t be effective until health care systems provide access to genetic testing and risk-reducing interventions at reasonable cost.

## 6 Impact of genetic testing on medical management

Both *BRCA1* and *BRCA2* encode proteins that are critical for homologous recombination DNA repair (34). Thus, homologous recombination repair is defective in breast cancers with germline P/LP *BRCA1* or *BRCA2* variants (35). Poly(adenosine diphosphate-ribose) polymerase (PARP) is an intracellular enzyme that helps repair damaged DNA. Thus, PARP inhibitors can selectively damage tumor cells that is deficient in homologous recombination repair (36, 37).

Several randomized controlled clinical trials investigated the role of PARP inhibitors in patients with P/LP *BRCA1* or *BRCA2* variants. Based on positive outcomes in two phase 3 trials; OlympiAD (38, 39) and EMBRACA (40, 41), two PARP inhibitors, olaparib and talazoparib, have been approved by the European Medicines Agency (EMA) the US Food and Drug Administration (FDA) for use in patients with P/LP BRCA-mutated, HER2-negative metastatic breast cancer. In the OlympiaAD trial, olaparib improved progression-free survival (PFS) compared with chemotherapy [7.0 months *vs* 4.2 months; hazard ratio (HR) for disease progression or death, 0.58; 95% CI, 0.43-0.80; P< 0.001)]. Talazoparib, in the EMBRACA trial, prolonged PFS (HR= 0.542, 95% CI, 0.413-0.711; P< 0.0001). A meta-analysis had shown that the efficacy, safety and tolerability of talazoparib and olaparib were found to be comparable (42). Another PARP inhibitor, veliparib, was tried in the BROCADE3 trial conducted in a similar patient population of P/LP *BRCA1*/2 with advanced HER2-negative breast cancer. The addition of veliparib to platinum-based doublet chemotherapy, led to significant and durable PFS improvement (43, 44).

PARP inhibitors were also shown to be effective when used in the adjuvant setting in patients with early stage high-risk HER2-negative breast cancer with P/LP *BRCA1*/2 variants; patients in the OlympiA trial receiving adjuvant olaparib had significantly longer overall survival (OS); 4-year OS was 89.8% in the olaparib group and 86.4% in the placebo group (95% CI, 0.1% - 6.8%) (45, 46). Based on the above data, PARP inhibitors are currently recommended by the NCCN guidelines for all patients with P/LP *BRCA1*/2 variants with recurrent or stage IV disease and to a select of patients with early-stage disease, **Table 2** (47).

**Table 2:** List of approved medications based on germline alterations.

Drug Approval Date (Reference)	Clinical indication	Clinical Outcome
Advanced-Stage Breast Cancer		
Olaparib January 12, 2018 (38, 39)	P/LP BRCA variants, HER2-negative Have been treated with chemotherapy either in the (neo)adjuvant, or metastatic setting.	Olaparib versus single agent palliative chemotherapy (capecitabine, eribulin or vinorelbine): ▪ PFS=7.0 versus 4.2 months (HR=0.58; 95% CI: 0.43–0.80), P<0.001 ▪ Median OS with olaparib: 19.3 months compared to 17.1 months for those treated with physician’s choice (HR= 0.90, 95% CI, 0.66-1.23), P = 0.513
Talazoparib October 16, 2018 (40, 41)	P/LP BRCA variants, HER2-negative	Talazoparib compared to single agent chemotherapy (capecitabine, eribulin, gemcitabine or vinorelbine), after a median follow-up of 11.2 months: ▪ PFS = 8.6 months with talazoparib versus 5.6 months with chemotherapy (HR for disease progression or death= 0.54; 95% CI, 0.41-0.71), P <.001 ▪ Median overall survival: 2.3 months (95% CI, 18.1-26.2) versus 19.5 months (95% CI, 16.3-22.4). HR for death= 0.76, P = 0.11
Veliparib (43, 44)	P/LP BRCA variants, HER2-negative	The addition of veliparib (versus placebo) to platinum-based doublet chemotherapy. Median follow-up: 35.7 months in the veliparib group and 35.5 months in the control group. ▪ Median PFS: 14.5 months (95% CI, 12.5-17.7) in the veliparib group versus 12.6 months (10.6-14.4) in the control group (HR= 0.71, 95% CI, 0.57-0·88), P=0·0016 ▪ Median OS: 32.4 months vs 28.2 months (HR=0.916, 95% CI, 0.736-1.140), P= 0.434
Early-Stage Breast Cancer		
Olaparib March 11, 2022 (45, 46)	P/LP BRCA variants, high-risk, HER2-negative Have been treated with at least 6 cycles of (neo)adjuvant chemotherapy containing anthracyclines, taxanes, or both.	▪ iDFS at 3 years: Olaparib: 86% (95% CI, 82.8-88.4) Placebo: 77% (95% CI, 73.7-80.1), P<0.0001 ▪ Overall Survival at 3 years : Olaparib: 75 (8%) deaths Placebo: 109 (12%) deaths, (HR= 0.68; 95% CI, 0.50-0.91), P=0.0091

P/LP, Pathogenic/Likely pathogenic; OS, Overall survival; PFS, Progression-free survival; iDFS, invasive disease-free survival; HR, Hazard ratio; CI, Confidence interval.

iDFS was defined as the time from randomization to date of first recurrence as invasive loco-regional, distant recurrence, contralateral invasive breast cancer, new cancer, or death from any cause.

## 7 International collaboration

Many researchers and scientists believe that germline genetic data can be viewed as a national commodity and can be considered a commercial enterprise. De-identified data should be made public and shared across the globe. However, international rules and regulations should be established at a global level to regulate the research conduct and ensure no deviations from the set goals. It’s anticipated that such data may help advance cancer research, accelerate discoveries, precision medicine and innovation at diagnostic, prognostic and therapeutic levels.

Such data sharing is more important in societies like ours, the population of which is underrepresented in the international genetic library and associated clinical trials (48). As an institution, through our journey to establish our program, we teamed up with internationally recognized centers including, MD. Anderson Cancer Center, Leeds cancer center and Princess Margert Hospital in Toronto.

## 8 Challenges

### 8.1 Awareness

Genetic counseling and testing, early detection, prevention, treatment and risk-reduction strategies in patients with pathogenic *BRCA1/2* variants are not sufficiently known by most patients and family members. More importantly, knowledge of primary health care providers and “general” surgeons who deal with high number of breast cancer patients in their first encounter is often suboptimal (49–51). Following the full integration of our clinical cancer genetics program, detailed family history is fully integrated in patients’ assessment (52). To enhance referral to genetic testing and counselling, appropriate national guidelines, linked to action plans, are highly needed.

### 8.2 The stigma

Patients may experience constant anxiety about the genetic test results, associated cancer diagnosis and genetic transmission to their children. Fears of being stigmatized by friends, relatives, and partners may push high-risk individuals to refuse to be tested, and when tested might not share positive results with their at-risk family members. Through proper counselling, such fears might be lessened by experienced medical staff.

### 8.3 Confidentiality

The confidentiality of doctor-patient relationship was never an issue; however, confidentiality of medical records, being electronic, was highly considered a potential hazard. Access to medical records is granted to all health care workers and leak of information, though strictly prohibited by the law, is a possibility. During our initial phases of germline genetic testing, and to encourage patients to undergo testing, results were not entered into electronic records and were kept literally in a “safe closet”. However, as we gained experience with the counselling process and gained patients’ confidence and to improve communicating testing results that may affect patients’ medical and surgical management across different services, results are now made available in medical records similar to all other laboratory testing.

### 8.4 Financial coverage

There is no clear guidance within the national healthcare legislation and governmental insurance in Jordan regarding the genetic testing. Testing is not available in any of the MOH or military (Royal Medical Services) hospitals. Germline genetic testing, however, was done at one of the university hospitals as part of a research project (53). Patients treated in the private sector may elect to do the test, at their own cost, as almost all private insurance providers don’t cover for testing and its associated preventive strategies.

## 9 Risk-reducing surgeries

### 9.1 Contralateral mastectomy

Breast cancer patients with *BRCA1*/2 pathogenic variants are at higher risk for contralateral breast cancer. Risk-reducing mastectomy among such patients showed a significant reduction in contralateral disease and some studies showed a significant improvement in the risk of breast cancer-related death (54–56).

### 9.2 Bilateral mastectomy

Many studies, including two meta-analyses, showed that prophylactic bilateral mastectomy reduces the risk for breast cancer in unaffected women with *BRCA1* [relative risk (RR)= 0.134, 95% CI, 0.019–0.937] and *BRCA2* (RR= 0.183, 95% CI, 0.072–0.468) (54, 57). However, the effect of such prophylactic surgery on survival is debatable. Only one of the above cited meta-analyses showed improved survival. Extent of surgery can be as aggressive as total mastectomy, though less aggressive surgeries including skin-sparing mastectomy (SSM) and nipple-sparing mastectomy (NSM), both associated with better cosmoses, are considered safe (58).

No clear recommendation on the age of risk-reducing surgery (RRM), however, given the increasing risk of breast cancer with age, it’s recommended to consider women age, preference, life expectancy and family history including age of onset. Access to these procedures is limited, and such procedures for “unaffected” carriers are not covered by public or private insurance.

### 9.3 Salpingo-oophorectomy

In addition to reducing the risk of ovarian cancer, risk-reducing salpingo-oophorectomy (RRSO) may have beneficial effect on breast cancer risk reduction; more so when done at younger age (59).

One prospective, multicenter study included 2482 women with *BRCA1* or *BRCA2* P/LP variants from 22 centers in Europe and North America to assess the relationship of risk-reducing mastectomy or salpingo-oophorectomy with cancer outcomes. In addition to lowering the risk of ovarian cancer, RRSO resulted in significant reduction in new breast cancer cases; compared with women who did not undergo RRSO. Women who underwent RRSO had a lower risk of first diagnosis of breast cancer in *BRCA1* mutation carriers; 20% *vs* 14% (HR= 0.63, 95% CI, 0.41-0.96) and *BRCA2* mutation carriers; 23% *vs* 7% (HR= 0.36, 95% CI, 0.16-0.82). Additionally, women who underwent RRSO had lower all-cause mortality, compared to those who didn’t; 10% *vs* 3% (HR= 0.40, 95% CI, 0.26-0.61), breast cancer-specific mortality; 6% *vs* 2% (HR= 0.44, 95% CI, 0.26-0.76), and ovarian cancer-specific mortality; 3% *vs* 0.4% (HR= 0.21, 95% CI, 0.06-0.80) (60).

In a meta-analysis that included 13,965 *BRCA1* and 7,057 *BRCA2* mutation carriers enrolled on 14 observational studies, the breast cancer risk among *BRCA1* mutation carriers was lowered by RRSO (HR = 0.63, 95% CI, 0.49-0.81, P < 0.01) and *BRCA2* mutation carriers (HR= 0.51, 95% CI, 0.34-0.75, P < 0.01). This risk reduction was more apparent among younger patients and was apparent in the immediate 5 years after surgery in both *BRCA1* and *BRCA2* mutation carriers (61).

These findings should encourage physicians to recommend RRSO for *BRCA1* and *BRCA2* carriers while young, however not before they reach the age of 40-44 years, to allow them to finalize their family planning, if they wish to.

## 10 Conclusions

Breast cancer germline genetic testing, as part of a more comprehensive program, is a national priority. In addition to its valuable impact in preventing additional cancer diagnoses in patients themselves and at-risk relatives, identification of P/LP variants may inform clinical management and choice of therapy among affected patients. Access to such testing is improving, however, it needs to be done in an organized way that ensures appropriate counselling of patients and family members. Risk-reducing strategies, including prophylactic surgeries, when needed, should be addressed by the national health care system, and a highly needed cancer control program.
